# Screening and Identification of Biomarkers in Ascites Related to Intrinsic Chemoresistance of Serous Epithelial Ovarian Cancers

**DOI:** 10.1371/journal.pone.0051256

**Published:** 2012-12-10

**Authors:** He Huang, Yujie Li, Jihong Liu, Minghui Zheng, Yanling Feng, Kunhua Hu, Yongwen Huang, Qidan Huang

**Affiliations:** 1 Department of Gynecology Oncology, Sun Yat-Sen University Cancer Center, State Key Laboratory of Oncology in South China, Guangzhou, P. R. China; 2 Department of Clinical Laboratory, The Sun Yat-sen Memorial Hospital of Sun Yat-sen University, Guangzhou, P. R. China; 3 Proteomics Center, Zhongshan School of Medicine, Sun Yat-sen University, Guangzhou, P. R. China; University of Texas Health Science Center at San Antonio, United States of America

## Abstract

**Objective:**

The ability to predict responses to chemotherapy for serous epithelial ovarian cancer (EOC) would be valuable since intrinsically chemoresistant EOC patients (persistent or recurrent disease within 6 months) gain little benefit from standard chemotherapy. The aim of this study was to screen and identify distinctive biomarkers in ascites of serous EOC associated with intrinsic chemoresistance.

**Methods:**

Protein samples from ascites of 12 chemosensitive and 7 intrinsically chemoresistant serous EOC patients were analyzed using two-dimensional fluorescence difference in gel electrophoresis (2-D DIGE) coupled with matrix-assisted laser desorption/ionization time-of-flight mass spectrometry (MALDI-TOF/TOF MS). Furthermore, the identified proteins were validated by ELISA in ascites samples from 19 chemosensitive and 9 intrinsically chemoresistant EOC patients.

**Results:**

The number of spots detected in all 2-D DIGE gels ranged from 1523–1711 using DeCyder software analysis. Thirty-four spots were differentially expressed based on the criteria of an average ratio of more than 1.5 and a student t-test *P* value <0.05. After MALDI-TOF/TOF MS analysis, 11 differentially expressed proteins, including 3 up-regulated and 8 down-regulated proteins, in ascites of chemoresistant tumors were successfully identified. Of the four selected proteins (ceruloplasmin, apoliprotein A-IV, transthyretin and haptoglobin) in ascites tested by ELISA, only ceruloplasmin was present at significantly different levels between the chemoresistant and chemosensitive ascites samples with average concentrations of 192.2 µg/ml and 157.5 µg/ml, respectively (*P* = 0.001).

**Conclusion:**

The significantly up-regulated level of ceruloplasmin in the ascites fluid of intrinsic chemoresistant serous EOC patients suggests its potential as a prognostic biomarker for responses to chemotherapy. This finding prompts further investigation with a larger study in order to validate the clinical utility of ceruloplasmin.

## Introduction

Platinum-based combination chemotherapy is the standard first-line treatment for advanced stage epithelial ovarian carcinoma (EOC). The tumors are considered “platinum sensitive” if the clinical progression-free interval is more than 6 months, but approximately 20% to 30% of patients progress or their tumors rapidly become resistant to this treatment [1]. These patients with intrinsic chemoresistance who experience a recurrence within 6 months gain little benefit from standard treatment. There is also evidence suggesting that the longer the interval until recurrence, the better the response rate to subsequent chemotherapy [2]. Therefore, chemoresistance for ovarian cancers may be present at the outset of treatment (intrinsic resistance) or may develop during treatment (acquired resistance).

Currently, chemoresistance of EOC can only be determined retrospectively after patients have experienced the burden and toxicity of ineffective therapy. Therefore, identification of characteristic molecular biomarkers related to intrinsic chemoresistance in EOC may lead to individually customized therapeutics and improvement of outcomes since standard chemotherapy affords them very little benefit.

Several recent studies have used gene microarrays to identify distinct gene expression in intrinsic chemoresistant ovarian cancer patients on different platforms, such as nylon cDNA arrays, Affymetrix chips and Agilent oligonucleotide microarrays [3], [4]. These studies have identified different prognostic and predictor genes which can distinguish early from late relapse or disease progression. However, transcription of a target gene in the tumor may not be a good predictor of drug resistance and prognosis for ovarian cancer. For example, mRNA abundance may not correlate with the corresponding protein expression and function. Furthermore, for some primary or recurrent ovarian cancer patients, tissue samples are not always available for gene profiling.

Unlike with other pelvic/abdominal malignant metastasis, massive ascites are a distinctive clinical manifestation in advanced EOC, with more than 80% of these patients having widespread metastasis to the serosal surfaces and associated peritoneal and/or pleural effusions [5]. Body fluids have been shown to be excellent media for biomarker discovery in cancer, and ascites fluid contains malignant epithelial cells and activated mesothelial cells, which can produce cytokines, growth factors and invasion-promoting components associated with invasion and metastasis [6]. This fluid therefore contains the secretome of ovarian cancer cells and reflects other microenvironmental factors of the malignancy.

Thus, applying the ever advancing technique of proteomics to the analysis of ascites may facilitate discovery of novel biomarkers that are more sensitive and specific than those currently available. The aim of our study was to screen and identify distinctive biomarkers in ascites of ovarian cancer associated with intrinsic chemoresistance by two-dimensional fluorescence difference in gel electrophoresis (2D-DIGE) technology, which would help identify these patients with poor prognosis and improve their clinical outcome with alternative therapies.

## Materials and Methods

### Ethics Statement

This study was approved by the Ethical Committee of Sun Yat-sen University Cancer Center Institutional Review Board. Written informed consent was obtained from each patient.

### Patients and Sample Preparation

In total, 19 ascites samples for 2D-DIGE and 28 cases of ascites for ELISA validation were collected during the period of Feb. 1, 2009 to Dec. 31, 2010 from intact serous EOC patients who underwent satisfactory cytoreductive surgery. Women with a previous cancer history and those receiving neoadjuvant chemotherapy were excluded. Cytoreductive surgery was performed via an abdominal midline incision. Samples of 5 ml ascites were obtained and stored at 80°C in liquid nitrogen prior to total hysterectomy, bilateral salpingo-oophorectomy, omentectomy and resection of all visible and palpable bulky tumor and lymphadenectomy, according to the National Comprehensive Cancer Network (NCCN) guidelines. Information on treatment and response was obtained by patient chart review.

After debulking, the patients received six cycles of platinum-based combination chemotherapy. The chemotherapy drugs included paclitaxel (135–175 mg/m^2^), carboplatin (area under curve [AUC] 5–6), doxepaclitaxel (70 mg/m^2^) and cisplatin (65–75 mg/m^2^). Based on the NCCN guidelines, intrinsically chemoresistant tumors were defined as those with persistent or recurrent disease within 6 months after the initiation of first-line platinum-based combination chemotherapy. Chemosensitive tumors were classified as those with a complete response to chemotherapy and a platinum-free interval of >6 months.

Ascites were centrifuged at 2,000 rpm for 15 min at 4°C to separate the fluid from cellular components. The suspension was briefly sonicated, and the debris was centrifuged at 14,000 rpm for 10 min at 4°C. The supernatant was resuspended and washed three times in ice-cold Tris-buffered sucrose solution (10 mM Tris, 250 mM sucrose, pH 7.0) and then scraped and lysed in ice-cold lysis buffer (30 mM Tris-HCl, 7 M urea, 2 M thiourea, 4% w/v CHAPS, pH 8.5).

Ascites samples were processed using the ProteoPrep Blue Albumin Depletion Kit (Sigma, St. Louis, MO, USA) that selectively removes albumin and IgG according to the manufacturer’s instructions. To purify the protein extraction and determine the final protein concentration, the 2-D Clean-up Kit (GE Healthcare, Buckinghamshire, UK) and 2-D Quant Kit (GE Healthcare) were used sequentially.

### Study Design and Protein Sample Labeling with CyDye

Twelve chemosensitve samples were divided equally into two subgroups with six samples each, and seven chemoresistance samples were likewise allocated into two subgroups with four or three samples each. Equal amounts of the protein samples in the same subgroup were mixed and separated into four equal aliquots (50 µg each). Two of the chemosensitive protein sample aliquots were labeled with Cy3, and two of the chemoresistant sample aliquots were labeled with Cy5. The remaining two chemosensitive samples were then labeled with Cy5 and the other two chemoresistant samples with Cy3. A sample consisting of equal amounts of all samples was used as the pooled internal standard (50 µg) and labeled with 200 pmol of Cy2. Therefore, one chemosensitive patient pool (Cy3 or Cy5), one chemoresistant patient pool (Cy5 or Cy3) and one internal standard (Cy2) were run in each gel, with four gels in total based on our design. This dye swapping strategy was adopted to avoid dye bias and allowed for equal distribution of Cy dyes in both patient groups.

Protein labeling was conducted with CyDye DIGE Fluors (GE Healthcare) as described in the Ettan DIGE user manual. Briefly, after incubating on ice for 30 min in the dark, 1 mL of 10 mM lysine was added to stop the reaction. For each gel, Cy2-, Cy3- and Cy5-labeled proteins (50 µg each) were mixed and adjusted to 450 µL with rehydration buffer [7 M urea, 2 M thiourea, 4% (w/v) CHAPS, 40 mM DTT, 1% IPG buffer (pH 4–7), 0.002% (w/v) bromophenol blue].

*2D-DIGE*. After rehydration, the labeled protein mixture for each gel was applied to an Immobiline DryStrip (24 cm, pH 4–7; GE Healthcare). Isoelectric focusing (IEF) was performed with an Ettan IPGphor II apparatus (GE Healthcare) as follows: 30 V for 12 hours, 500 V for 1 hour, 1,000 V for 1 hour and 10,000 V for up to a total of 85,000 Volt-hour. After IEF, the proteins were reduced and alkylated by successive 15 min treatments with equilibration buffer containing 2% (w/v) DTT, followed by 2.5% (w/v) iodoacetamide. The proteins were then resolved in 12.5% SDS-PAGE gels using an Ettan DALTsix instrument (GE Healthcare). In order to facilitate MS analysis, an unlabeled pool protein sample (500 µg) was run in parallel on a preparative gel and stained with Deep Purple Total Protein Stain (GE Healthcare) according to the manufacturer’s instructions.

### Gel Image Acquisition and Analysis

Gel images were acquired on a Typhoon 9400 scanner (Amersham Biosciences) and analyzed using DeCyder Software (V6.0, GE Healthcare) as described previously [7]. The Cy2, Cy3 and Cy5 signals were individually imaged with excitation/emission wavelengths of 488/520, 532/580 and 633/670 nm, respectively. Preparative gels (Deep Purple Total Protein Stain) were scanned with excitation/emission wavelengths of 532/560 nm according to the user’s manual. Proteins in chemosensitive ascites samples were compared with those in chemoresistant ones. Increases or decreases of protein abundance of more than 1.5-fold (t-test and ANOVA, *P*<0.01) were considered significant changes. The corresponding protein spots were selected in the stained preparative gel for spot picking.

### Protein Spot Handling

The selected protein spots in the preparative gels were automatically picked and handled in an Ettan Spot Handling Workstation (GE Healthcare). The selected protein spots were washed with 15 mM ammonium bicarbonate and 50% methanol and then digested in 0.02 µg/mL sequencing grade trypsin solution (Promega, Madison, WI, USA) at 37°C for 2 h. The tryptic peptides were extracted with 50% (v/v) acetonitrile (ACN) and 0.5% (v/v) trifluoroacetic acid (TFA), dissolved in 5 mg/mL R-cyano-4-hydroxycinnamic acid (Amersham Bioscience) in 50% (v/v) ACN and 0.1% (v/v) TFA and then spotted on the MS sample plate.

### Matrix-assisted Laser Desorption/ionization Time-of-flight Mass Spectrometry (MALDI-TOF/TOF MS) Analysis

Protein identification was performed with the ABI 4800 Proteomics MALDI-TOF/TOF Analyzer (Applied Biosystems, Foster City, CA, USA) in positive ion reflector mode. Monoisotopic peak masses were acquired in a range of 900–4,000 Da with a signal-to-noise ratio (S/N) >200. Trypsin autolytic peptides of masses 842.5 and 2211.1 were used as internal standards. Five of the most intense ion signals were automatically selected as precursors for MS/MS acquisition, excluding the trypsin autolysis peaks and matrix ion signals. The peptide mass fingerprint (PMF) combined MS/MS spectra were searched against the NCBInr database using GPS Explorer™ software (Version 3.6, Applied Biosystems) and MASCOT version 2.1 (Matrix Science). The search parameters were set as follows: *Homo sapiens*, trypsin cleavage (one missed cleavage allowed), carbamidomethylation as fixed modification, methionine oxidation as variable modification, peptide mass tolerance set at 75 ppm and fragment tolerance set at 0.2 Da. A significantly high MASCOT score that resulted in a confident interval (CI) greater than 95% for PMF or MS/MS data for a spot was considered as a credibly identified protein. The other criteria included a minimum of four peptides hits in PMF data-based identification and at least two peptides with distinct sequences identified in MS/MS analysis.

### ELISA Validation

Ascites samples (5 mL) were centrifuged at 2,000 rpm for 15 min at 4°C to separate the fluid from cellular components. The suspension was briefly sonicated, and the debris was centrifuged at 14,000 rpm for 10 min at 4°C. The supernatant was resuspended and stored at −80°C.

ELISA assay kits for each of the analytes selected for further analysis were purchased from Abcam. These analytes were as follows: apoliprotein A-IV (Apo-AIV), ceruloplasmin, transthyretin and haptoglobin. Assays were performed following the instructions of the kit. Briefly, the color change due to the enzyme-substrate reaction of each well in the microtiter plate was measured spectrophotometrically at a wavelength of 450 nm. The concentration of each tested protein in the sample was then determined by comparing the optical density (OD) to that of the standard curve.

Student’s t-test was conducted to compare differences between serum protein values. The SPSS 16.0 software package (SPSS, Chicago, IL, USA) was used to conduct the statistical analyses, and a two-tailed *P* value of less than 0.05 was considered statistically significant.

## Results

### Clinical Patient Information

Nineteen ascites samples of serous EOC patients were analyzed using 2D-DIGE to screen potential biomarkers associated with differential responses to chemotherapy. Samples from a separate cohort of 28 patients with serous EOC were used for validation of the 2D-DIGE results by ELISA. All patients had received satisfactory cytoreductive surgery. There were no significant differences in age at diagnosis, tumor differentiation and International Federation of Gynecology and Obstetrics (FIGO) staging between the patients in the chemosensitive and chemoresistant groups. Demographic and clinical features of the cases are shown in Table 1.

**Table 1:** Clinical characteristics of study subjects.

No	Age	Differentiation	FIGOstage	Regimen after primary surgery	Evaluation*	DIGE	ELISA Validation
1	50	Moderate	IIIb	Paclitaxel/carboplatin	Sensitive	(+)	(+)
2	54	Poor	IIIc	Paclitaxel/carboplatin	Sensitive	(+)	(+)
3	38	Well	IIc	Paclitaxel/carboplatin	Sensitive	(+)	(+)
4	58	Poor	IIIc	Paclitaxel/cisplatin	Sensitive	(+)	(+)
5	51	Poor	IIIc	Doxepaclitaxel/carboplatin	Sensitive	(+)	(+)
6	55	Moderate	IIIb	Paclitaxel/cisplatin	Sensitive	(+)	(+)
7	60	Poor	IIIc	Doxepaclitaxel/carboplatin	Sensitive	(+)	(+)
8	33	Poor	IIIc	Doxepaclitaxel/carboplatin	Sensitive	(+)	(+)
9	42	Poor	IVa	Paclitaxel/cisplatin	Sensitive	(+)	(+)
10	60	Poor	IIIc	Paclitaxel/carboplatin; Doxepaclitaxel/carboplatin**	Sensitive	(+)	(+)
11	49	Moderate	IIb	Paclitaxel/carboplatin	Sensitive	(+)	(+)
12	56	Poor	IIIc	Paclitaxel/carboplatin; Paclitaxel/cisplatin***	Sensitive	(+)	(+)
13	49	Poor	IIIc	Paclitaxel/carboplatin	Sensitive	(−)	(+)
14	44	Well	IIIc	Doxepaclitaxel/cisplatin	Sensitive	(−)	(+)
15	59	Poor	IIIb	Paclitaxel/carboplatin	Sensitive	(−)	(+)
16	57	Moderate	IVa	Paclitaxel/carboplatin; Doxepaclitaxel/carboplatin**	Sensitive	(−)	(+)
17	69	Poor	IIIc	Paclitaxel/cisplatin	Sensitive	(−)	(+)
18	52	Poor	IIIc	Paclitaxel/carboplatin	Sensitive	(−)	(+)
19	60	Poor	IIIa	Doxepaclitaxel/carboplatin	Sensitive	(−)	(+)
20	58	Moderate	IIIc	Paclitaxel/carboplatin	Resistance	(+)	(+)
21	63	Poor	IIIc	Paclitaxel/carboplatin	Resistance	(+)	(+)
22	66	Poor	IIIb	Doxepaclitaxel/carboplatin; Doxepaclitaxel/cisplatin***	Resistance	(+)	(+)
23	64	Moderate	IIIc	Paclitaxel/cisplatin	Resistance	(+)	(+)
24	56	Poor	IIIb	Doxepaclitaxel/carboplatin	Resistance	(+)	(+)
25	45	Poor	IIIc	Paclitaxel/carboplatin; Doxepaclitaxel/carboplatin**	Resistance	(+)	(+)
26	60	Poor	IIIc	Paclitaxel/carboplatin	Resistance	(+)	(+)
27	41	Poor	IIIc	Paclitaxel/cisplatin	Resistance	(−)	(+)
28	66	Moderate	IIIc	Doxepaclitaxel/carboplatin	Resistance	(−)	(+)

* Resistance: intrinsically chemoresistant tumors were defined as those with persistent or recurrent disease within 6 months after the initiation of first-line platinum-based combination chemotherapy. Sensitive: chemosensitive tumors were classified as those with a complete response to chemotherapy and a platinum-free interval of >6 months.

** Paclitaxel was changed to doxepaclitaxel due to grade III neurotoxicity.

*** Carboplatin was changed to cisplatin due to grade III neutropenia.

**Figure 1:**
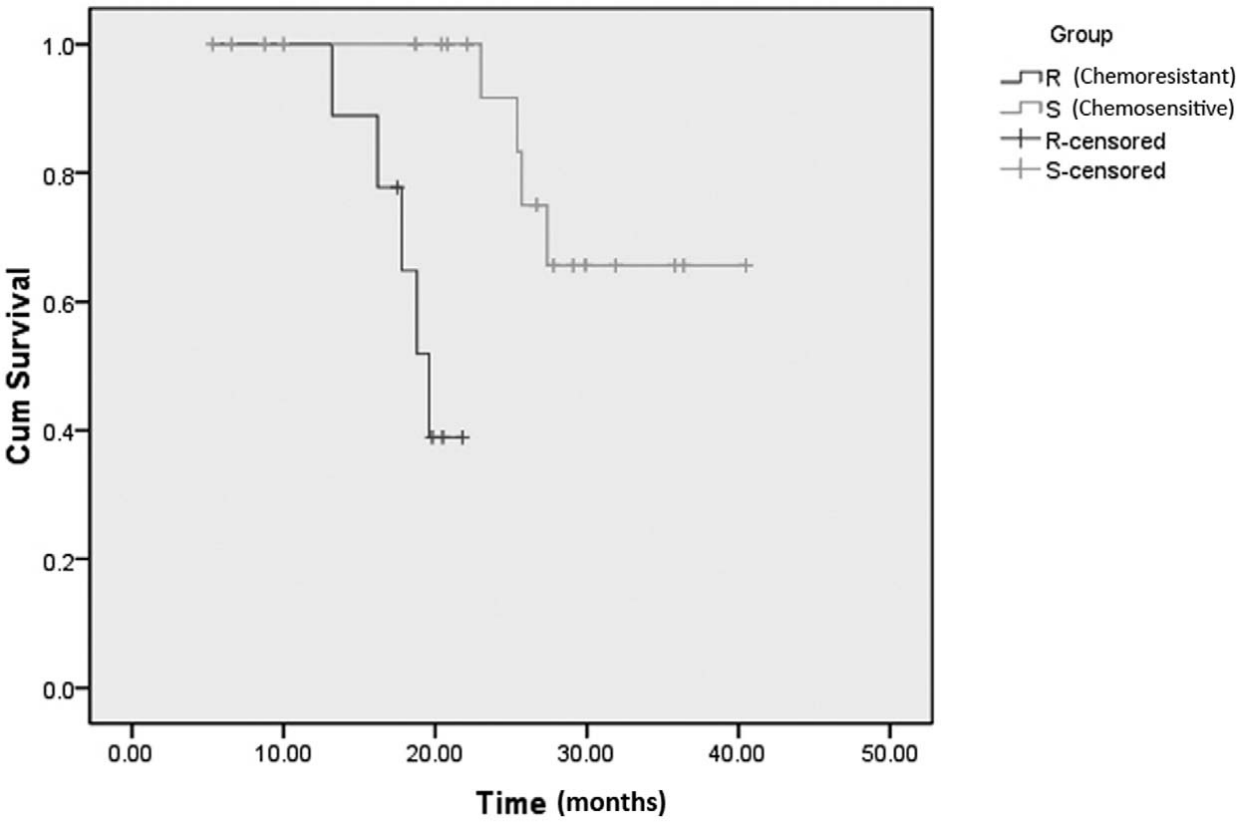
Comparison of survival between chemosensitive and intrinsic chemoresistant serous EOC patients. A significant difference (*P*  =  0.007) was observed in survival, which favored patients with chemosensitive tumors.

In addition, survival rates of the 28 patients tested by ELISA were compared according to their different responses to chemotherapy. By March 2012, four of the nine patients (44.4%) in the chemoresistant group and three of nineteen patients (15.8%) had died in the chemosensitivity group. The median survival time of the nine chemoresistant ovarian cancer patients in our study was 18.9 months. However, a longer period of follow-up was needed to determine an accurate median survival of chemosensitive patients, which was more than 18.9 months. Based on the observation period in this study, the difference in survival between the two groups as observed using Kaplan–Meier estimates was significant (*P* = 0.007), favoring those with better responses to chemotherapy (Fig. 1).

### 2D-DIGE Analysis and MS/MS Identification

**Figure 2:**
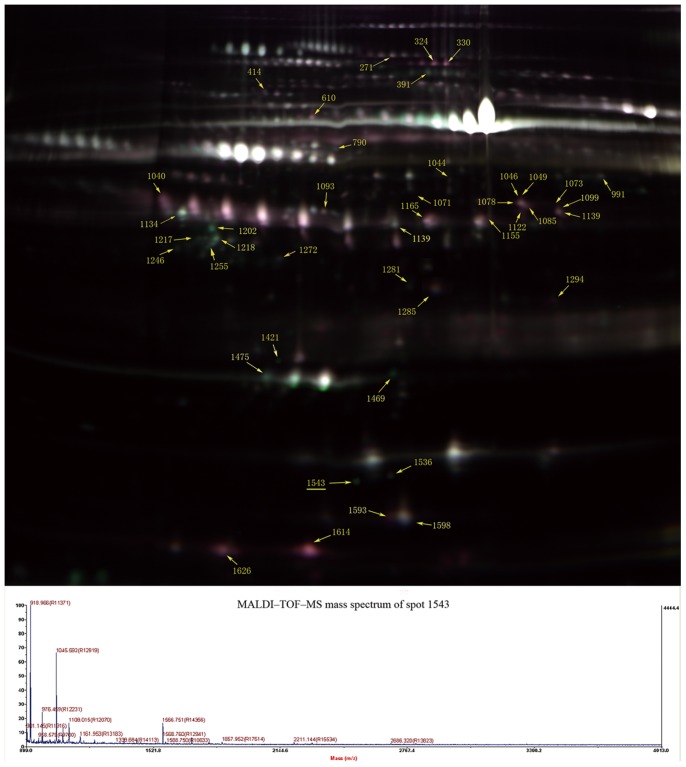
(top) Proteomic analysis of ascites by 2D-DIGE. A representative 2D-DIGE image (merged image) showing the protein profile of ascites of chemosensitive and chemoresistant ovarian cancer patients labeled with Cy5 (red spots) and Cy3 (green spots), respectively, with an internal standard labeled with Cy2. IPG strips (24 cm, pH 4-7) were used for IEF prior to standard SDS-PAGE (12.5% polyacrylamide) for the second dimension. The molecular weight range in the vertical dimension is approx from 150 to 10 kD. Proteins identified as differentially expressed are indicated with yellow arrows with assigned numbers from the DeCyder analysis. The numbers in the figure correspond to those presented in Table 2; (bottom) MALDI–TOF–MS mass spectrum of spot 1543 identified as ceruloplasmin according to the matched peaks.

According to our experimental design described in Materials and methods, four 2D-DIGE gels in total were set up for proteomic analysis of chemoresistant versus chemoresistant patient ascites. For each gel a merged image was generated from three images of the chemosensitive, chemoresistant and internal standard samples. A representative DIGE gel showing the overlay of the Cy2, Cy3 and Cy5 labeled images is shown in Figure 2.

A total of 1523 to 1711 spots were detected in different Differential In-gel Analysis (DIA) workspaces in all the gels using DeCyder software. In the Biological Variation Analysis (BVA) module, the Cy3 image from gel number four was chosen as the master gel as it had the maximum number of spots. Thirty-four spots were found to be differentially expressed based on the criteria of having an average ratio of more than +1.5 or less than −1.5 and a student t-test *P* value <0.05. Among them, 14 spots were found to be down-regulated in the chemoresistant ascites, and 27 were up-regulated compared to the chemosensitive patients.

Some of the differential spots detected by DeCyder software could not be visualized in the preparative gel stained with colloidal coomassie likely due to low abundance. After visual review, 20 protein spots showing high abundance and significantly altered expression in chemoresistant versus chemosensitive patients were selected for MALDI-TOF/TOF MS analysis. A total of 11 differentially expressed proteins, including 3 up-regulated and 8 down-regulated proteins, in ascites of chemoresistant tumors compared with chemosensitive tumors were successfully identified. The peptide count of the identified proteins varied from 4 to 33. Fold changes of levels of the 11 identified proteins in the two groups along with the detailed Mascot search results are given in Table 2.

**Table 2:** Differentially expressed proteins between chemosensitive and chemoresistant ovarian cancer ascites identified by MALDI-TOF/TOF MS.

Spot No^a^	Protein name	Ratio^b^	Accession no^c^	Mascot Score^d^	Peptide count	MW/PI (kDa)	Protein functions^e^
1093	Apolipoprotein A-IV Precursor	−1.57	gi|93163358	628	33	45.4/5.28	Antioxidants, lipid transport and metabolism, acute-phase protein, tumor^f^
1281	ACTB protein	−1.69	gi|15277503	430	17	40.1/5.55	Microtubules, movement of the cell, cytoskeletal protein
1285	Chain A, TTR Variants	−1.56	gi|2098257	355	8	13.9/5.35	Transthyretin variants implicate formation of amyloid fibril
1421	Apolipoprotein A-I preproprotein	2.16	gi|4557321	389	17	30.8/5.56	Lipid transport and metabolism, lecithin cholesterol acyltransferase, acute-phase protein
1475	Proapolipoprotein	2.7	gi|178775	307	18	29.0/5.45	Recombinant proapolipoprotein A-I
1536	Ceruloplasmin CpF5	−1.66	gi|223092	151	4	18.7/5.31	Histidine-rich proteolytic fragment of ceruloplasmin
1543	Ceruloplasmin	2.86	gi|1620909	79	6	11.6/5.43	Carrier Proteins, oxidoreductases, acute-phase protein, tumor^f^
1593	Transthyretin	−2.87	gi|48145933	121	4	16.0/5.5	Thyroid hormone transport, serine proteinase inhibitors, acute-phase protein, tumor^f^
1598	Covalent Dimer Of Transthyretin	−2.17	gi|55669575	442	10	12.8/5.33	Affects the amyloid pathway
1614	Haptoglobin Hp2	−2.27	gi|223976	318	6	42.3/6.23	Isoform of haptoglobin
1626	Haptoglobin	−2.6	gi|1212947	289	4	39.0/6.27	Recycling of heme- iron, acute-phase protein, Mucoproteins, tumor^f^

a Spot no. assigned by DeCyder 2-D Differential Analysis Software V6.0. corresponding to the DIGE image in Figure 1.

b Decreased or increased ratio of protein in ascites of intrinsic chemoresistant ovarian cancer compared to chemosensitive ones; “–” indicates down-regulation of protein in ascites.

c Accession no. from NCBInr database.

d Mascot scores greater than 40 were considered significant.

e Protein function mainly categorized according to MeSH Tree Structure.

f Alterations in the serum level may indicate: occurrence, tumor progression, response to therapy or prognosis for some malignancy reported in the literature.

Several proteins were identified in multiple spots. For example, spots 1421, 1593, and 1598 were each identified as the transthyretin protein, which is a transport and acute phase protein, or its variants. Similarly, spots 1614 and 1626 were identified as haptoglobin, while spots 1536 and 1543 were determined to be ceruloplasmin and its proteolytic fragment. The identified proteins are involved in four different aspects of cell function and are related to tumor progression and metastasis (one cytoskeletal protein, three energy/lipid metabolism proteins, three carrier proteins and four acute-phase proteins).

### ELISA Validation of Apo-AIV, Ceruloplasmin, Transthyretin and Haptoglobin

To validate the results of the proteomic analysis, we determined the levels of four proteins including Apo-AIV, ceruloplasmin, transthyretin and haptoglobin in ascites samples from 19 chemosensitive and 9 intrinsically chemoresistant EOC patients by ELISA. Based on previous proteomic profiles and considering our purpose of screening unique biomarkers in the ascites, cytoskeletal protein (ACTB) was excluded from this analysis.

The ELISA results confirmed our MALDI-TOF/TOF MS findings in that the level of ceruloplasmin was significantly higher in chemoresistant than in chemosensitive ascites. The average concentration of ceruloplasmin was 157.5 µg/ml in the chemosensitive group and 192.2 µg/ml in the chemoresistant group (*P* = 0.001), while the levels of Apo-AIV, transthyretin and haptoglobin were not significantly different between the two groups. These results are summarized in Table 3.

**Table 3:** Concentrations of proteins in ascites of intrinsically chemoresistant and chemosensitive EOC.

Analytes	Chemosensitive	Chemoresistant	*P* value
Apo-AIV (µg/mL)	230.3±41.4	215.0±35.7	0.349
CP (µg/mL)	157.5±11.5	192.2±19.5	0.001
HB (mg/L)	263.1±38.0	266.6±44.1	0.832
TTR (mg/L)	316.2±35.6	337.9±36.5	0.146

* Apo-AIV, apoliprotein A-IV; CP, ceruloplasmin; HB, haptoglobin; TTR, transthyretin.

## Discussion

The prognosis of ovarian cancer is known to be strongly associated with the length of the platinum-free interval from the primary first-line platinum-based combination chemotherapy treatment to relapse. The longer this interval lasts, the better the response rate to subsequent chemotherapy [8]. Thus, standard chemotherapy is largely non-beneficial for intrinsically chemoresistant ovarian cancer patients (with persistent or recurrent disease within 6 months). The ability to predict the response to standard chemotherapy in these patients would be extremely valuable in allowing the early use of individualized therapeutics to help prolong survival and avoid unnecessary side effects of ineffective treatments.

We have noted that many advanced stage ovarian cancer patients present with rapid growth of intraperitoneal tumors along with abdominal distention as a result of accumulation of ascites fluid in the peritoneal cavity. Mechanistically, ascites formation occurs as malignant cells secrete proteins, growth factors and cytokines that cause neovascularization, angiogenesis, increased fluid filtration and/or lymphatic obstruction, resulting in the buildup of serum-like fluid within the abdomen [9], [10]. The rich medium provides support for malignant cells to proliferate and further metastasize despite the lack of matrix substrata, allowing these cells to overcome the apoptosis associated with loss of attachment. This implies that malignant cells and mesothelial cells in ascites up-regulate survival signals in order to persist in the hypoxic but otherwise rich liquid milieu [11]. Thus, ascites is an excellent reservoir for the identification of useful cancer biomarkers, especially in EOC patients [12].

In a study by Kislinger’s group, ascites were separated into cellular and fluid fractions, followed by mass spectrometry analysis of each fraction [13]. While over 2,500 proteins within ascites were identified, only 229 proteins were found in the fluid fraction. After integrated computational analysis of the ascites proteome combined with proteomic data from human plasma and urine microarray data sets and protein–protein Interaction Database I2D, 80 candidate serological ovarian cancer biomarkers were selected for further validation. Kuk and colleagues also carried out proteomic analysis of ascites fluid based on multiple separation and fractionation techniques [14]. A total of 52 proteins were selected from 445 unique proteins in the ascites fluid as good candidates for ovarian cancer biomarkers in future investigations. These authors all found proteomic analysis to be a significant resource for ovarian cancer research and a framework for biomarker discovery.

To our knowledge, proteomic studies of ovarian cancer ascites are limited, and a comparative study between intrinsic chemoresistant and chemosensitive ovarian cancer ascites by DIGE technology has not been previously reported. The findings of our study may aid in the prediction of therapeutic responses and disease prognosis for ovarian cancer patients. Our first aim was to find unique biomarkers just in the ascites fluid and not in the cell fraction as in Kislinger’s study [13]. Thus, we separated the fluid fraction from cellular components for 2D-DIGE by centrifugation. As biomarkers may be present at low concentrations in body fluids such as serum and ascites, one major challenge to performing in-depth analysis of proteomes by mass spectrometry is the presence of highly abundant proteins such as albumin and immunoglobulins, which constitute 65–97% of serum proteins [15]. These abundant proteins limit the ionization efficiency during MS analysis, preventing the identification of low abundance proteins. Therefore, depleting the highly abundant proteins by using a 2D-clean up kit was necessary before our analysis in order to detect lowly abundant biomarkers.

In our study, a total of 11 differentially proteins were identified between chemosensitive and chemoresistant ovarian cancer ascites. It was not unexpected that some proteins were identified in multiple spots, since many proteins in ascites are known to exist as isoforms. For example, haptoglobin and transthyretin were represented by two and three spots, respectively, and in both cases, the individual spots were similarly down-regulated. Additionally, the same protein being present in several different spots could have represented biologically relevant modifications or proteolytic fragments (e.g., ceruloplasmin).

The change in serum concentration of ceruloplasmin, along with many other acute phase proteins identified among the differentially expressed proteins, implicated the presence of immune and inflammatory responses caused the tumor. The results were not unexpected since different inflammatory mediators, which play diverse roles such as inducing angiogenesis, invasion, autocrine growth loops and resistance to apoptosis, are elevated in ovarian carcinoma [16]. Many acute phase proteins such as haptoglobin and transthyretin have also been recently characterized as ovarian cancer biomarkers for early detection [17]. Moreover, in a previous gene profiling study, Bachvarov and colleagues found that down-regulated genes in chemosensitive serous EOC tumors included numerous genes involved in lipid metabolism and transport, inflammation, as well as genes known to enhance tumor progression and invasion [18]. These findings implicated acute phase proteins as candidate biomarkers of interest for further investigation.

Ceruloplasmin, a plasma glycoprotein that transports copper throughout the body, was the only protein confirmed by ELISA in the current study to be differentially expressed in the ascites between the chemoresistant and chemosensitive patients in this study. Interestingly, high serum levels of ceruloplasmin have been demonstrated in various cancers such as thyroid, prostate and colon cancer [19], and microarray analysis has linked this gene to tumor invasion and metastasis in breast cancer [20]. Altered serum ceruloplasmin levels after treatment (chemotherapy or radiation) have been observed in many patients with malignancies, such as laryngeal, cervical and breast cancers. Evidently, a more significant decrease of the serum ceruloplasmin level after treatment is linked with a better response to therapy, as these alterations may influence disease outcome [21], [22]. These previous observations support our finding that the concentration of ceruloplasmin was significantly lower in the ascites fluids of chemosensitive ovarian cancer patients.

Roles for ceruloplasmin have been suggested in cancer-related processes, including angiogenesis and neovascularization. The protein also serves as a surrogate marker for total body copper. Therefore, the lower serum ceruloplasmin level in our study may be secondary to the deficiency in total body copper associated with tumor suppression. In a study by Cox et al., tetrathiomolybdate (TM), a copper chelator was used to reduce body stores of copper in a murine model of head and neck squamous cell carcinoma (SCC) established using the highly aggressive SCC VII/SF cell line [23]. The authors found that as the total body copper was reduced by TM, the serum ceruloplasmin level was proportionately reduced, with the baseline level decreasing from by28%. As significantly suppressed levels of both the growth of SCC and tumor vascularity were identified, their results suggested a potential efficacy of TM in the treatment of cancers via its effects on angiogenesis and neovascularization. Similar results were seen in a phase II trial with advanced kidney cancer patients in which the anti-tumor effects of TM (decreased vascularity and tumor mass) were associated with lower serum copper and ceruloplasmin levels [24].

Thus, the change in serum concentration of ceruloplasmin may indicate that it is an acute phase protein secreted in response to the oxidative stress in inflammation associated with the tumor and/or that it is secondary to the deficiency of total body copper. Our analysis was based on primary serous EOC tumors without mixed histotypes of ovarian tumors, or recurrent and metastatic tumors. To our knowledge, this is the first reported proteomic analysis by 2D-DIGE analysis of ascites from patients with intrinsic chemoresistant and chemosensitive ovarian cancer. Additionally, the results may help to predict therapeutic responses and provide disease prognosis as well as new clues into the mechanism of chemoresistance for ovarian cancer. However, there are possible biases in our study. As mentioned before, the expression of ceruloplasmin may be associated with tumor progression. Therefore, the high ceruloplasmin level in ascites in our study may be caused by relatively advanced tumor metastasis associated with worse prognosis. Additionally, biases may be caused by serum components in the ascites fluid or even from our exclusion of the ascites samples mixed with blood due to tumor bleeding.

In our study, the number of patients was limited by the length of time required for collection of samples. An ascites sample of serous ovarian adenocarcinoma was taken during the primary surgery before chemotherapy, and then we waited six months after six cycles of chemotherapy to determine the status of each patient as chemosensitive or chemoresistant. Therefore, longitudinal studies with a larger number of ascites samples are needed for further validation of the utility of ceruloplasmin as a biomarker. Although it may be challenging to determine the proper combination, identifying multiple predictive biomarkers will be more informative.

In conclusion, the concentration of ceruloplasmin was found to be significantly higher in the ascites fluids of intrinsic chemoresistant serous EOC patients, compared with those from chemosensitive patients. Although further validation with more ascites samples is needed to evaluate its utility, this finding suggests that ceruloplasmin is a potential prognostic biomarker for EOC patient responses to chemotherapy.
